# Risk factors for the development of neonatal sepsis in a neonatal intensive care unit of a tertiary care hospital of Nepal

**DOI:** 10.1186/s12879-021-06261-x

**Published:** 2021-06-09

**Authors:** Sulochana Manandhar, Puja Amatya, Imran Ansari, Niva Joshi, Nhukesh Maharjan, Sabina Dongol, Buddha Basnyat, Sameer M. Dixit, Stephen Baker, Abhilasha Karkey

**Affiliations:** 1 Oxford University Clinical Research Unit, Patan Academy of Health Sciences, Kathmandu, Nepal; 2 Centre for Tropical Medicine and Global Health, Medical Sciences Division, Nuffield Department of Medicine, University of Oxford, Oxford, UK; 3 Department of Pediatrics, Patan Academy of Health Sciences, Patan Hospital, Kathmandu, Nepal; 4 Centre for Molecular Dynamics Nepal, Kathmandu, Nepal; 5 Cambridge Institute of Therapeutic Immunology & Infectious Disease (CITIID) Department of Medicine, University of Cambridge, Cambridge, UK

**Keywords:** Neonatal sepsis, NICU, Nepal, Risk factors, MDR, ESBL, Etiology, Epidemiology

## Abstract

**Background:**

Sepsis is an overwhelming and life-threatening response to bacteria in bloodstream and a major cause of neonatal morbidity and mortality. Understanding the etiology and potential risk factors for neonatal sepsis is urgently required, particularly in low-income countries where burden of infection is high and its epidemiology is poorly understood.

**Methods:**

A prospective observational cohort study was conducted between April 2016 and October 2017 in a level three NICU at a tertiary care hospital in Nepal to determine the bacterial etiology and potential risk factors for neonatal sepsis.

**Results:**

Among 142 NICU admitted neonates, 15% (21/142) and 32% (46/142) developed blood culture-positive and -negative neonatal sepsis respectively. *Klebsiella pneumoniae* (34%, 15/44) and *Enterobacter* spp. (25%, 11/44) were the most common isolates. The antimicrobial resistance of isolates to ampicillin (100%, 43/43), cefotaxime (74%, 31/42) and ampicillin-sulbactam (55%, 21/38) were the highest. *Bla*_TEM_ (53%, 18/34) and *bla*_KPC_ (46%, 13/28) were the commonest ESBL and carbapenemase genes respectively. In univariate logistic regression, the odds of sepsis increased with each additional day of use of invasive procedures such as mechanical ventilation (OR 1.086, 95% CI 1.008–1.170), umbilical artery catheter (OR 1.375, 95% CI 1.049–1.803), intravenous cannula (OR 1.140, 95% CI 1.062–1.225); blood transfusion events (OR 3.084, 95% CI 1.407–6.760); NICU stay (OR 1.109, 95% CI 1.040–1.182) and failure to breast feed (OR 1.130, 95% CI 1.060–1.205). Sepsis odds also increased with leukopenia (OR 1.790, 95% CI 1.04–3.082), increase in C-reactive protein (OR 1.028, 95% CI 1.016–1.040) and decrease in platelets count (OR 0.992, 95% CI 0.989–0.994). In multivariate analysis, increase in IV cannula insertion days (OR 1.147, 95% CI 1.039–1.267) and CRP level (OR 1.028, 95% CI 1.008–1.049) increased the odds of sepsis.

**Conclusions:**

Our study indicated various nosocomial risk factors and underscored the need to improve local infection control measures so as to reduce the existing burden of sepsis. We have highlighted certain sepsis associated laboratory parameters along with identification of antimicrobial resistance genes, which can guide for early and better therapeutic management of sepsis. These findings could be extrapolated to other low-income settings within the region.

**Supplementary Information:**

The online version contains supplementary material available at 10.1186/s12879-021-06261-x.

## Background

Neonatal sepsis refers to the clinical syndrome of a systemic inflammatory response, which develops secondarily to a proven or suspected infection in neonates [1]. While the incidence of sepsis ranges from 1 to 5 cases/1000 live-births in high income countries (HICs), it is estimated to be at least 3 to 20 fold higher in low- and middle- income countries (LMICs) [2]. In 2018, there were 2.5 million neonatal deaths globally, with the majority occurring in Sub-Saharan Africa and South Asia; 15% of these deaths were associated with sepsis [3]. In Nepal, neonatal sepsis is common and of the12,881 neonatal deaths that occurred in 2015, 18.4% were attributed to sepsis [4].

Immature immunity, preterm birth and very low birth weight (< 1500 g) place neonates at increased risk of infections. Many vulnerable newborns often require special clinical care and admission to a neonatal intensive care unit (NICU). Ironically, due to their unique vulnerability coupled with hostile environmental factors of NICUs, admission to an NICU is in itself a risk factor for the development of hospital acquired infections (HAIs). Studies have shown that NICUs have a greater incidence of infections compared to other neonatal units [5, 6]. Various invasive modalities of therapeutic/supportive clinical care used in NICUs such as parenteral feeding, mechanical ventilation, and intravascular catheterization have been shown to be associated with increased risk of neonatal sepsis [6].

Most of the published studies from Nepal on neonatal sepsis are cross-sectional and/or retrospective using routine microbiology or NICU data and primarily addressing the bacterial and clinical characteristics only. Longitudinal developmental studies aiming at rigorously observing the neonates throughout their stay in NICU would provide unprecedented opportunities to collect real time data on predisposing factors associated with neonatal sepsis, along with other valuable information such as clinical-pathological characteristics, bacterial etiology and associated antimicrobial resistance (AMR). Understanding of such variables that are reflective of the local scenario can provide crucial information required for early diagnosis and effective treatment in addition to developing best fitted preventive strategies. Such studies are scarce in Nepal. Here, in this prospective cohort observational study, we aimed to fulfill this gap by longitudinally following up the neonates admitted to NICU in a tertiary care hospital of Nepal in order to determine the clinical-pathological features, potential risk factors, bacterial etiology and antimicrobial resistance profile of neonatal sepsis.

## Methods

### Ethical approval

Before carrying out the study, an ethical clearance was taken from Nepal health research council (NHRC278/2015) and Oxford Tropical Research Ethics Committee (OxTREC 24–16). All methods were performed following the ethical principles as stated by the Declaration of Helsinki and in accordance to Good Clinical and Laboratory Practices.

### Setting and study design

This prospective observational cohort study was conducted in a level three NICU of Patan Hospital, a tertiary care referral teaching hospital of the Patan Academy of Health Sciences, located in the Lalitpur Metropolitan area of the Kathmandu valley. The eight bedded NICU has a provision for admitting only those intensive care requiring neonates that are born in Patan Hospital. Within NICU, two beds are separated for a dedicated ICU care of culture positive neonates. Neonates born elsewhere are admitted to the pediatric intensive care unit (PICU). Neonates without risk factors for sepsis are admitted to a clean nursery, while those having underlying risk factors for, or evidence of infection are sent to the septic nursery for observation and non-intensive medical care.

### Enrollment and follow up of the neonates

The parents/guardians of the neonates who were admitted to the NICU from April 2016 till October 2017 were approached for informed consent to take part in the study. The consented neonates were enrolled and followed up on daily basis for development of any signs of septicemia throughout their stay in the NICU or until one of the final outcomes (discharge, transfer to other department, death or left without medical advice) was attained. All relevant data on clinical, therapeutic and laboratory investigation were recorded in a pre-tested case report form (CRF) by referring to the clinical note marked by the pediatrician. Data were recorded onto CliRes Data Management System. The integrity of data entry was validated by double entry of the information by two different individuals.

### Inclusion and exclusion criteria

All consented neonates who were born in Patan hospital during a sampling period of 1.5 years from April 2016 to October 2017 and requiring admission to NICU were included in the study. Those presenting with gross congenital abnormalities and those admitted or readmitted to the NICU after being discharged from the hospital were excluded.

### Diagnosis of sepsis

Enrolled neonates were suspected of having sepsis based on clinical signs and/or underlying risk factors as listed in Additional file 1. Once suspected, relevant biological samples were collected for sepsis screening tests as given in Additional file 1 [7, 8]. Neonates were immediately treated with empirical antimicrobial therapy according to the neonatal treatment guidelines of Patan hospital (Additional file 2). As per the NICU protocol adopted by Patan hospital, sepsis was diagnosed as culture positive sepsis if the blood culture was positive in a patient with clinical signs of sepsis. Culture negative sepsis was diagnosed in the presence of clinical signs of sepsis but a negative blood culture. The overall workflow for sepsis diagnosis is outlined in Additional file 3. Neonates who were not suspected of sepsis during their entire stay in the NICU were designated as non-sepsis group. The neonates who were clinically suspected of sepsis earlier, but yielded negative results in both sepsis screen tests and culture, along with improved clinical signs were ruled out as sepsis cases and formed a part of the non-sepsis group. For statistical comparative analysis, the non-sepsis group was taken as the control group against the blood culture positive sepsis group to identify the risk factors for sepsis.

### Microbiological investigation

One to two ml of peripheral blood samples were drawn from the neonates clinically suspected of sepsis and before the administration of antimicrobials. The samples were aseptically injected into BD-BACTEC Peds Plus/F culture vials (Becton Dickinson, UK) and incubated in an automated BD BACTEC FX40 (Becton Dickinson, UK) culture system. Upon being flagged as culture positive, small volume of blood was aseptically aspirated with sterile syringe and inoculated on 5% sheep blood agar, MacConkey agar and chocolate agar. Bacterial identification was conducted by standard microbiological methods and antimicrobial susceptibility testing (AST) was performed using modified Kirby-Bauer disc diffusion method [9],with inhibition zone sizes interpreted according to Clinical Laboratory Standards Institute (CLSI) 2017 breakpoint guidelines [10]. Gram-negative bacilli (GNB) that were resistant (zone of inhibition ≤22 mm) or intermediate (zone of inhibition 23-25 mm) to cefotaxime (30 μg) were suspected as producing extended-spectrum beta-lactamases (ESBL).

### Confirmatory tests for ESBL and AmpC production

Phenotypic confirmatory tests for ESBL production were performed using the combination disc diffusion method [11] using a beta-lactam antimicrobial with and without a beta-lactamase inhibitor (D62C, D68C, Mast group Ltd., Liverpool, UK). The isolate was considered as ESBL producer if the size of zone of inhibition in beta-lactamase inhibitor supplemented disc was equal to or more than 5 mm as compared to the beta lactam alone. For genetic test, the bacterial DNA was extracted from an overnight culture of cryogenically preserved bacterial isolates by heating at 90 °C for 10 min, followed by centrifugation at 4000 rpm for 15 min. The supernatant was used as template DNA for PCR assay. Multiplex PCR was performed for detection of genetic markers associated with ESBL (*bla*_CTX-M-1, 2, 9, 8/25_ [12]_,_ *bla*_TEM_ [13]_,_ *bla*_SHV_ [14]_,_ *bla*_OXA_ [14]) and carbapenemase (*bla*_KPC_ [15], *bla*_NDM-1_ [16], *bla*_OXA-48_ [15], *bla*_OXA-23, 24, 51, 58_ [17]) mediated resistance (Additional file 4). Twenty-five microliter of PCR master mix containing 1XPCR buffer, 1 unit Hotstart Taq DNA polymerase (Qiagen, Germany),1.5 mM MgCl_2_, 0.2 mM dNTPs mix,0.1–0.2 μM of each primers and 3 μl crude template DNA were used for amplification. PCR conditions were 95 °C/15 min, followed by 35 cycles of 95 °C/40 s, annealing temperature of 52–61 °C/40 s and extension at 72 °C/50 s with final extension of 72 °C for 7 min. The PCR amplicons were electrophoresed in 1.5% agarose gel and results were interpreted based on the presence of expected product size as depicted by positive control.

### Statistical analysis

Statistical analyses were conducted in SPSS (version 20.0). The descriptive statistics of qualitative variables were expressed in absolute frequency (N) with percentage (%). That of quantitative variables was calculated in mean ± standard deviation (SD) or median with interquartile range (IQR). Odds ratio with 95% confidence interval (CI) was calculated under univariate logistic regression analysis. The significance threshold of *p* ≤ 0.05 was considered. Five independent variables that gave highly significant *p*-values (< 0.001) in univariate analysis were modeled using backward stepwise multiple logistic regression. In this process, only two variables remained significant at the final stage.

## Results

### Demographic features

From April 2016 to October 2017, there were a total of 11,779 live births in Patan hospital, of which 204 (1.73%) required admission to the NICU; 142 of these neonates were enrolled in this study. The demographic features of the enrolled neonates are shown in Additional file 5. Briefly, among the 142 enrolled neonates, 59% (83/142) were preterm and 65% (93/142) were male; 16% (22/142) were of very low birth weight (< 1500 g); 15% (22/142) and 18% (26/142) required intubation and resuscitation respectively immediately after birth. The majority of deliveries (68%, 96/142) were cesarean and respiratory distress was common (55%, 78/142). The use of various invasive and non-invasive devices of clinical care was frequent. The median duration of NICU stay and entire hospital stay were 7 days (IQR 4–12) and 16 days (IQR 10–26), respectively. The use of antimicrobials during the study period is shown in Additional file 2. Over 90% of 142 enrolled neonates received the first line antimicrobials (ampicillin and amikacin), while 56 and 26% of enrolled cases received cefotaxime and meropenem, respectively.

### The burden and types of sepsis

Sepsis was clinically suspected in 49% (70/142) of the enrolled neonates; while remaining 72 neonates didn’t develop any signs of sepsis during their NICU stay. Amongst total, 15% (21/142) and 32% (46/142) of enrolled neonates developed blood culture positive and negative sepsis respectively. Later, due to improved clinical condition and negative sepsis screen results, three of the clinically suspected cases were ruled out of sepsis, giving a total non-sepsis group of 75 neonates. The 21 culture positive neonates yielded a total of 44 blood culture positive sepsis events. Nine cases developed single sepsis episode, three neonates developed two culture positive events and rest nine cases developed more than three culture positive events during the study period. For multiple blood culture positive events caused by the bacteria of same genus and similar antimicrobial susceptibility profile, they could be either due to the persistence of earlier infection or re-infection by new bacterial strain. Eighty percent (35/44) of culture positive sepsis events were of late onset type (after 72 h of birth). Of total enrolled neonates, 14% (20/142) died during the study period; eight of those who died had sepsis.

### Microbiological results

A total of 118 blood samples were microbiologically investigated during the study period from the 70 enrolled neonates who were clinically suspected of sepsis. Of 118 blood samples, 44 samples were culture positive, giving a blood culture positive proportion of 37% (44/118). The majority (89%, 39/44) of bacterial isolates was Gram negative bacilli (GNB) with *Klebsiella pneumoniae*, *Enterobacter* spp. and *Acinetobacter* spp. being the most common, constituting 34% (15/44), 25% (11/44) and 18% (8/44) of the total isolates respectively. Gram positive cocci (GPC) were less common, with coagulase negative *Staphylococcus* (CoNS) being the most prevalent GPC of total isolates (9%, 4/44) (Table 1). As per the NICU protocol of Patan hospital, if the neonate fulfilled the clinical criteria of sepsis, the isolation of CoNS from neonatal blood samples is considered significant and the neonate is treated accordingly.

**Table 1:** The bacteriological profile of neonatal blood culture samples shown by number and percentage

Bacterial isolates	n	%
**GNB**		
*Klebsiella pneumoniae*	15	34.1
*Enterobacter* spp.	11	25.0
*Acinetobacter* spp.	8	18.2
*E. coli*	3	6.8
*Klebsiella oxytoca*	1	2.3
*Pseudomonas* spp.	1	2.3
**Total GNB**	**39**	**88.6**
**GPC**		
CoNS	4	9.1
*S. aureus*	1	2.3
**Total GPC**	**5**	**11.4**
**Total (All isolates)**	**44**	

*GNB* Gram negative bacilli, *GPC* Gram positive cocci, *CoNS* Coagulase negative *Staphylococcus*

The results of AST and ESBL confirmatory tests for the bacterial isolates from blood are shown in Tables 2 and 3, respectively. Briefly, 72% (31/43) of bacterial isolates were multi-drug resistant (resistant to three or more different classes of antimicrobial agents). Overall, 100 and 37% of isolates were resistant to ampicillin and amikacin respectively. Further, 74% (31/42), 55% (21/38) and 34% (13/38) of the isolates were resistant to cefotaxime, ampicillin-sulbactam and meropenem, respectively. In general, GNB were more susceptible to amikacin, ciprofloxacin and cotrimoxazole, while less susceptible towards various beta-lactam antimicrobials, except meropenem. Twenty one percent (7/34) of GNB isolates were phenotypically confirmed to be ESBL producers, of which *Klebsiella* spp. contributed 40% (6/15). Overall, *bla*_TEM_ (53%, 18/34) and *bla*_KPC_ (46%, 13/28) were the most common ESBL and carbapenemase genes respectively in GNB. Ninety four percent (14/15) and 47% (7/15) of *Klebsiella* spp. had *bla*_TEM_ and *bla*_NDM-1_ respectively; 73% (8/11) of *Enterobacter* spp. had *bla*_KPC_ and 83% (5/6) of *Acinetobacter* spp. had *bla*_OXA-51_ resistance gene. All isolates tested negative for *bla*_IMP_ and *bla*_VIM_ genes.

**Table 2:** Antimicrobial resistance profile of bacteria isolated from blood samples, shown by proportion (percent)

GNB, n	MDR	AMP	AMK	GEN	CTX	CHL	CIP	OFX	TS	MEM	SAM	PTZ
*Klebsiella* spp., 16	12/16 (75)	16/16 (100)	8/16 (50)	8/16 (50)	13/16 (81)	3/16 (19)	8/16 (50)	5/16 (31)	8/16 (50)	4/16 (25)	10/16 (63)	5/16 (31)
*Enterobacter* spp., 11	9/11 (82)	11/11 (100)	1/11 (9)	3/11 (27)	8/11 (73)	8/11 (73)	2/11 (18)	1/11 (9)	2/11 (18)	6/11 (55)	5/11 (45)	4/11 (36)
*Acinetobacter* spp., 8	5/7 (71)	7/7 (100)	3/7 (43)	3/7 (43)	6/7 (86)	6/7 (86)	3/7 (43)	2/7 (29)	4/7 (57)	3/7 (43)	4/7 (57)	2/7 (29)
*E coli*, 3	0/3 (0)	3/3 (100)	0/3 (0)	0/3 (0)	0/3 (0)	0/3 (0)	0/3 (0)	0/3 (0)	0/3 (0)	0/3 (0)	1/3 (33)	0/3 (0)
*Pseudomonas aeruginosa*, 1	1/1 (100)	1/1 (100)	1/1 (100)	1/1 (100)	1/1 (100)	1/1 (100)	1/1 (100)	1/1 (100)	1/1 (100)	0/1 (0)	1/1 (100)	0/1 (0)
Total for GNB	**27/38 (71)**	**38/38 (100)**	**13/38 (34)**	**15/38 (39)**	**28/38 (74)**	**18/38 (47)**	**14/38 (37)**	**9/38 (24)**	**15/38 (39)**	**13/38 (34)**	**21/38 (55)**	**11/38 (29)**
GPC	**MDR**	**AMP**	**AMK**	**GEN**	**CTX**	**CHL**	**CIP**	**OFX**	**TS**	**ERY**	**CLI**	**OXA**
*Staphylococcus aureus*, 1	1/1 (100)	1/1 (100)	1/1 (100)	1/1 (100)	1/1 (100)	0/1 (0)	1/1 (100)	1/1 (100)	0/1 (0)	1/1 (100)	1/1 (100)	1/1 (100)
CoNS, 4	3/4 (75)	4/4 (100)	2/4 (50)	3/4 (75)	2/3 (67)	1/4 (25)	3/4 (75)	2/3 (67)	1/1 (100)	3/3 (100)	0/3 (0)	2/3 (67)
Total for GPC	**4/5 (80)**	**5/5 (100)**	**3/5 (60)**	**4/5 (80)**	**3/4 (75)**	**1/5 (20)**	**4/5 (80)**	**3/4 (75)**	**1/2 (50)**	**4/4 (100)**	**1/4 (25)**	**3/4 (75)**
Total for GNB and GPC	**31/43 (72)**	**43/43 (100)**	**16/43 (37)**	**19/43 (44)**	**31/42 (74)**	**19/43 (44)**	**18/43 (42)**	**12/42 (29)**	**16/40 (40)**			

*GNB* Gram negative bacilli, *GPC* Gram positive cocci, *CoNS* Coagulase negative *Staphylococcus*, *MDR* Multi-drug resistant, *AMP* Ampicillin, *AMK* Amikacin, *GEN* Gentamycin, *CTX* Cefotaxime, *CHL* Chloramphenicol, *CIP* Ciprofloxacin, *OFX* Ofloxacin, *TS* Cotrimoxazole, *MEM* Meropenem, *SAM* Ampicillin-Sulbactam, *PTZ* Piperacillin-Tazobactam, *ERY* Erythromycin, *CLI* Clindamycin, *OXA* Oxacillin

**Table 3:** Results of confirmatory tests for ESBL and carbapenemase production, shown by proportion (percent)

Bacterial isolates^d^	Phenotypic test		Molecular (PCR) test^a^							
	ESBL	AmpC	***bla***_**TEM**_	***bla***_**CTXM-1**_^**b**^	***bla***_**SHV**_	***bla***_**NDM-1**_	***bla***_**OXA**_	***bla***_**KPC**_	***bla***_**OXA-48**_	***bla***_**OXA-51**_^**c**^
*Klebsiella* spp	6/15 (40)	1/15 (7)	14/15 (94)	7/15 (47)	5/15 (33.4)	7/15 (47)	6/15 (40)	5/15 (33)	7/15 (46.7)	NA
*Enterobacter* spp.	1/11 (9)	1/11 (9)	4/11 (36)	2/11 (18)	0/11 (0)	0/11 (0)	4/11 (36)	8/11 (73)	3/11 (27.2)	NA
*Acinetobacter* spp.	0/6 (0)	0/6 (0)	0/6 (0)	0/6 (0)	0/6 (0)	0/6 (0)	0/6 (0)	NA	NA	5/6 (83)
Grand Total	**7/34 (21)**	**`2/34 (6)**	**18/34 (53)**	**9/34 (26)**	**5/34 (15)**	**7/34 (21)**	**10/34 (29)**	**13/28 (46.4)**	**10/28 (36)**	**5/6 (83)**

*NA* not tested

^a^All tested isolates were negative for *bla*_IMP_ and *bla*_VIM_

^b^All tested isolates were negative for other *bla*_CTXM_ family genes: *bla*_CTXM-2_, *bla*_CTXM-8_, *bla*_CTXM-9_, *bla*_CTXM-25_

^c^All *Acinetobacter* spp. isolates tested negative for other *bla*_OXA_ genes: *bla*_OXA-23_, *bla*_OXA-24_, *bla*_OXA-58_

^d^Both *E. coli* isolates were negative for all AMR genes tested

### Risk factors associated with the occurrence of neonatal sepsis in NICU

In order to identify potential predictors of sepsis in our setting, odds ratio was calculated by univariate logistic regression for various neonatal, maternal, laboratory and environmental factors (Table 4). The analysis was based on 75 non-sepsis and 21 culture proven sepsis cases. - Of several origins of potential exposure factors analyzed, majority of the factors that stood out to be statistically significant were found to be related to the nosocomial exposures. Overall, we found that every single day increase in the use of various invasive devices like mechanical ventilation (OR 1.086, 95% CI 1.008–1.170, *p* = 0.030), UAC (OR 1.375, 95% CI 1.049–1.803, *p* = 0.021), UVC (OR 1.325, 95% CI 1.047–1.676, *p* = 0.019), IV cannula (OR 1.140, 95% CI 1.062–1.225, *p* < 0.001),OG tube (OR 1.612, 95% CI 1.038–2.503, *p* = 0.033) and every additional blood transfusion events (OR 3.084, 95% CI 1.407–6.760, *p* = 0.005) were associated with increased odds of sepsis development in the univariate logistic regression analysis. It was also found that every additional day of stay in NICU and hospital increased the risk of sepsis development with odds ratios of 1.109 (95% CI 1.040–1.182, *p* = 0.002) and 1.097 (95% CI 1.031–1.167, *p* = 0.004) respectively. Similarly, with every single day increase in failure to feed orally (breast and spoon feeding) and conversely, enteral feeding increased the risk of neonatal sepsis with odds ratios of 1.130 (95% CI 1.060–1.205, *p* < 0.001), 1.140 (95% CI 1.064–1.222, *p* < 0.001) and 1.163 (95% CI 1.059–1.278, *p* = 0.002) respectively. In laboratory parameters, when compared to normal WBC count range (7000–30,000/ μl), leukopenia (< 7000 WBC/μl) was associated with increased odds of sepsis (OR 1.790, 95% CI 1.04–3.082, *p* = 0.036). Similarly, decrease in platelets count and increase in C-reactive protein level raised the odds of sepsis with odds ratios of 0.992 (95% CI 0.989–0.994, *p* < 0.001) and 1.028 (95% CI 1.016–1.040, *p* < 0.001) respectively. In multivariate analysis, increase in IV cannula insertion days (OR 1.147, 95% CI 1.039–1.267, *p* = 0.006) and CRP level (OR 1.028, 95% CI 1.008–1.049, *p* = 0.006) increased the odds of sepsis.

**Table 4:** Logistic regression analysis for determining odds ratio of potential risk factors for neonatal sepsis. The variable values for each group are also shown

Variables	Variable values mean (sd) or frequency (%)		Logistic regression analysis		
	Culture-positive neonates	Non-Sepsis neonates	Odds ratio	95% CI	***p***-value
**Univariate analysis**					
***Obstetric-peripartum features:***					
Maternal age, years	29.2 (sd 5.5)	27.6 (sd 4.9)	1.062	0.964–1.168	0.221
Labor pain, hours	4.3 (sd 2.2)	5.7 (sd 3.7)	0.828	0.552–1.242	0.363
Vaginal test, times	0.7 (sd 0.5)	0.8 (sd 0.9)	0.955	0.595–1.53	0.848
PPROM:					
Absence	15 (70%)	60 (80%)	1	Reference	
Presence	6 (30%)	15 (20%)	1.714	0.564–5.208	0.342
Induction of labor:					
Not induced	16 (76%)	59 (79%)	1	Reference	
Induced	5 (24%)	16 (21%)	1.133	0.356–3.566	0.832
Delivery mode:					
Normal	5 (24%)	17 (23%)	1	Reference	
Vaginal/assisted	2 (9%)	8 (11%)	0.85	0.135–5.366	0.863
C-section	14 (67%)	50 (67%)	0.952	0.298–3.037	0.934
Amniotic fluid color:					
Clear	20 (95%)	63 (84%)	1	Reference	
Meconium stained	1 (5%)	12 (16%)	0.263	0.032–2.158	0.214
Suctioning at birth:					
No	15 (72%)	38 (50%)	1	Reference	
Yes	6 (28%)	37 (50%)	0.4	0.14–1.144	0.087
Resuscitation at birth:					
No	16 (76%)	60 (80%)	1	Reference	
Yes	5 (24%)	15 (20%)	1.25	0.395–3.958	0.704
Intubation at birth:					
No	16 (76%)	62 (83%)	1	Reference	
Yes	5 (24%)	13 (17%)	1.49	0.463–4.796	0.503
***Neonatal features:***					
Birth weight, grams:					
> 2500 (Normal)	6 (29%)	35 (47%)	1	Reference	
1500–2500 (Low)	10 (47%)	31 (41%)	1.882	0.613–5.777	0.269
< 1500 (Very low)	5 (24%)	9 (12%)	3.241	0.803–13.072	0.098
Gestational age, weeks	34.4 (sd 3.8)	35.8 (sd 3.6)	0.902	0.792–1.0278	0.121
Gender:					
Female	9 (43%)	26 (35%)	1	Reference	
Male	12 (57%)	49 (65%)	0.707	0.264–1.897	0.492
Lethargy, days	11.6 (sd 6.8)	6.6 (sd 5.3)	1.139	1.046–1.24	**0.003**
***Neonatal laboratory features***:					
WBC count (×10^3/μl):					
Normal (7–30)	12.5 (sd 6.2)	14.7 (sd 6.2)	1	Reference	
Leukopenia (< 7)	4.8 (sd 6.2)	4.9 (sd 6.4)	1.79	1.040–3.082	**0.036**
Leukocytosis (> 30)	32 (sd 13.8)	41.8 (sd 10.2)	0.154	0.019–1.216	0.076
Platelets (×10^3/μl)	129 (sd 130)	234 (sd 114.7)	0.992	0.989–0.994	**0.000**
CRP level (mg/dl)	72.0 (sd 72.5)	14.8 (sd 28.1)	1.028	1.016–1.040	**0.000**
***Therapeutic/supportive care features:***					
Transfusion, times	4.7 (sd 4.1)	1.5 (sd 0.6)	3.084	1.407–6.76	**0.005**
UAC insertion, days	26.5	17.6	1.375	1.049–1.803	**0.021**
UVC insertion, days	31.2	19.9	1.325	1.047–1.676	**0.019**
IV cannula, days	19.6 (sd 8.4)	11.1 (sd 6.8)	1.14	1.062–1.225	**0.000**
Foley’s insertion, days	9.8 (sd 7.1)	7.5 (sd 5.2)	1.064	0.956–1.184	0.257
Intubation, days	11 (sd 8.1)	6.6 (sd 6.2)	1.086	1.008–1.170	**0.03**
OG tube insertion, days	15.5	8.1	1.612	1.038–2.503	**0.033**
NG tube insertion, days	2.7	2.2	1.07	0.972–1.177	0.168
***Neonatal feeding features:***					
Not breast fed, days	17.1 (sd 9.6)	8.6 (sd 6.5)	1.13	1.060–1.205	**0.000**
Not spoon fed, days	16.7 (sd 8.5)	8.5 (sd 6.4)	1.14	1.064–1.222	**0.000**
Enteral fed, days	9.3 (sd 7.2)	4.7 (sd 4.1)	1.163	1.059–1.278	**0.002**
***Outcome:***					
NICU stay, days	16.1 (sd 9.3)	8.9 (sd 6.7)	1.109	1.040–1.182	**0.002**
Hospital stay, days	23.9 (sd 10.1)	14.9 (sd 9.1)	1.097	1.031–1.167	**0.004**
Final outcome:					
Survived			1	Reference	
Dead	4 (22%)	12 (18%)	1.333	0.369–4.817	0.661
**Multivariate analysis**					
IV cannula insertion, days			1.147	1.039–1.267	**0.006**
CRP level (mg/dl)			1.028	1.008–1.049	**0.006**

*CRP* C-reactive protein, *IV* Intravenous, *NG* Naso-gastric, *OG* Oro-gastric, *PPROM* Prolonged premature rupture of the membrane, *sd* standard deviation, *UAC* Umbilical artery catheter, *UVC* Umbilical vein catheter

Significant *p*-values are shown in bold fonts

## Discussion

In this prospective longitudinal cohort study on NICU admitted neonates, we found a high burden of sepsis (15% culture positive and 32% culture negative sepsis), 80% of culture positive sepsis events being of late onset type and 89% being caused by GNB, with *Klebsiella pneumoniae* being the most common (34%, 15/44) isolates. Nearly three quarters of the bacterial isolates were MDR and nearly half of the isolates contained the ESBL and carbapenemase genes *bla*_TEM_ and *bla*_KPC_. Further, we found that every additional day of insertion of various invasive devices, failure to oral feeding and stay in NICU as well hospital increased the odds of sepsis development.

The burden of neonatal sepsis may vary within a setting by time and between settings depending on the differences in local epidemiology of sepsis. Also, probably due to differences in study design and definitions, the prevalence of culture positive sepsis reported from various neonatal units in South Asia varied widely from 6 to 57%, compared to 15% in this study [18–22]. We found the majority (80%) of culture proven episodes to be of late onset type suggesting horizontal transmission to be main mode of infection, as compared to other Asian countries [23, 24]. Alternatively, studies from Pakistan and Bangladesh reported higher prevalence of early onset sepsis suggesting more vertical transmission or poor hygienic procedure during delivery [19, 25].

In this study, GNB were the most common (87%, 39/44) cause of both early and late onset sepsis, which corresponds to findings in several LMICs [26, 27]. Among all isolates, *Klebsiella pneumonia* was the most common isolate, similar to observations in other NICUs [25, 26]. In fact, this bacteria has been a predominantly persisting etiologic agent of sepsis outbreaks in Patan hospital NICU requiring intermittent shut down of the unit [28, 29]. Increasing AMR of clinical Enterobacteriaceae isolates is a global public health threat [30]. Due to the extensive use of broad spectrum antimicrobials, NICUs are likely to play a major role in the emergence and spread of MDR organisms [31]. In our study, 72% of isolates were MDR and higher proportion of GNB isolates were resistant to third generation cephalosporins (72%) and meropenem (34%), as reported in several NICUs of LMICs [27, 32, 33]. This is in contrary to studies from other regions like Southeast Asia [23], Africa [34] and Latin America [35],where below 10% of isolates were resistant to carbapenems despite higher proportion of resistance to extended spectrum cephalosporins. Beta-lactam antimicrobials are the mainstay for therapeutic management of sepsis. Higher percentage of AMR phenotypes of GNB and possessing genetic determinants conferring potentially transferable resistance to all available beta-lactams portrays a formidable therapeutic challenge. We found that nearly half of GNB isolates carried *bla*_TEM_ ESBL gene and *bla*_KPC_ carbapenemase genes; similar to that in some Indian studies [33, 36, 37].

Here, at least 80% of sepsis episodes were NICU acquired as they developed after 48 h of NICU admission. This is further corroborated by the facts that we enrolled only the inborne neonates who were never discharged from Patan hospital after birth and had no apparent signs of infection at the time of admission. This strongly suggests that these infections were acquired horizontally from the environment during their stay in NICU. Further, due to constrain of resources, a physically separated area was not available to entirely isolate the culture positive cases in our unit during the study period. Though two beds in NICU were dedicated for the care of culture positive neonates, being inside the same NICU unit, the sharing of human and medical resources could not be avoided. A proper aseptic cleaning of medical devices and proper hand hygiene was practiced as much as possible before sharing the resources between septic and non-septic neonates residing in the same unit. However, an absolute seclusion might not have achieved leading to massive cross infection events from infected to non-infected neonates. Thus, during the study period, the burden of infection was high in our unit and a majority of infections were environmentally acquired. This is a common scenario in several resource strapped hospitals of LMICs. In our study, we found that with every single day increase in the use of invasive devices such as mechanical ventilation, IV cannula, central vascular lines (UAC, UVC) and every additional event of blood transfusion, the odds of developing sepsis were increased by ratios of 1.09, 1.14, 1.37, 1.32 and 3.08 respectively. The use of umbilical catheterization [6, 38, 39] and mechanical ventilation [5, 39] were also significantly associated with sepsis in other studies. While invasive procedures are integral components of neonatal care in NICU, these life-sustaining devices often simultaneously serve as portals of systemic infections as observed in our study. Our findings thus emphasize the need to strengthen local infection control measures such as hand hygiene; and aseptic placement and maintenance procedures for invasive devices. Additionally, the removal of invasive devices whenever possible to reduce their dwelling time may be one of the factors in reducing the prevalence of sepsis. Here, we also found that every additional day of stay in NICU and hospital increased the risk of sepsis development with odds of 1.109 and 1.097 respectively, as observed in other studies [6, 40]. An increased stay in NICU and other hospital wards invariably raises the exposure to various nosocomial risk factors such as handling, device uses etc., thereby increasing the occurrence of sepsis. This consequently may increase other health complications and overall health care cost. Hence, it is suggested that the unnecessarily protracted NICU or hospital stay for the neonates should be avoided as far as possible to reduce the sepsis incidences.

Breast milk has been considered to have a protective effect against infections due to its anti-infective, microbiome-modulating, and immune-stimulatory properties [41–43]. In this study, we found that every single day increase in failure to breast feeding during the study period significantly increased the odds of sepsis with odds ratio of 1.13, as with failure to spoon feeding (OR 1.14). Conversely, each day increase in enteral feeding with OG tube during their NICU stay also increased the risk of sepsis development with an odds ratio of 1.163. Breast or spoon feeding may not be possible in some NICU admitted neonates due to several underlying conditions. However, our study suggests that shortening the duration of enteral feeding as much as possible and conversely switching to oral feeding whenever possible may help in reducing the occurrence of sepsis.

Besides being 1.5 years long and one of few prospective longitudinal studies from Nepal on neonatal sepsis, another strength of our study is the investigation of AMR genes, which is limited to phenotypic AMR profile only in other studies from Nepal. Given the increasing burden of MDR pathogens in NICUs, it becomes imperative to investigate pathogens for presence of AMR genes so that it aids in selecting optimal antimicrobial therapies. Further, our ability to convince the hospital administration to build a physically separated isolation room with NICU facility (NISO, Neonatal ICU Isolation room) based on the results of this study is one of the greatest achievements of our study. Since after the advent of NISO room which was developed soon following our study for handling the culture-positive neonates only, the incidence of sepsis has drastically reduced in our unit. Our study has few limitations too. We might not have captured a complete picture of the epidemiology and transmission dynamics of neonatal sepsis because the follow up of enrolled neonates was restricted only to the NICU irrespective of their further destiny (discharge or transfer to other hospital wards). Majority (86/142) of enrolled neonates were directly admitted to NICU soon after their birth. Depending on their prior clinical course, others were however admitted to NICU after being kept in nursery or mother’s ward postnatally. Though neonates did not have any apparent signs of infection at the time of NICU admission, we could not detect any incubating colonization or infection and thus could not assure that all observed infections were acquired solely from NICU. Further, fungal sepsis etiology, environmental source on infections and compliance to hand hygiene were not measured.

## Conclusions

Our study determined the burden; demographic, clinical and laboratory parameters; etiology; phenotypic and genetic profile of AMR, and risk factors for NICU linked neonatal sepsis in the low resource setting of Nepal. High burden of neonatal sepsis and associated antimicrobial resistance was documented. Further, it was evidenced that majority of sepsis episodes were late-onset and hospital acquired with the odds of sepsis being raised with prolong insertion several invasive devices of neonatal care. These findings can aid in an early identification of high-risk neonates and in selecting an optimal antimicrobial therapy in similar settings. It also emphasizes the need to conduct infection surveillance and improve infection control measures. The accommodation of extensive environmental investigation to pinpoint the source and interventional approach to further validate preventable risk factors are warranted in further studies.

## Supplementary Information

**Additional file 1.**

**Additional file 2.**

**Additional file 3.**

**Additional file 4.**

**Additional file 5.**

## Data Availability

All study data generated or analyzed during this study are available in this paper and its supplementary information files.

## Abbreviations

AMR: Antimicrobial resistance
AST: Antimicrobial susceptibility test
CI: Confidence interval
CLSI: Clinical Laboratory Standards Institute
CoNS: Coagulase-negative *Staphylococcus*
CRF: Case report form
CRP: C-reactive protein
ET: Tube Endo-tracheal tube
ESBL: Extended-spectrum beta-lactamase
GNB: Gram-negative bacilli
GPC: Gram-positive cocci
HICs: High income countries
HAIs: Hospital acquired infections
ICU: Intensive care unit
IV: Intravenous cannula
IQR: Intra-quartile range
LMICs: Low and middle income countries
MDR: Multi-drug resistant
NG tube: Noso-gastric tube
NHRC: Nepal health research council
NICU: Neonatal intensive care unit
OG tube: Oro-gastric tube
OR: Odds ratio
OxTREC: Oxford Tropical Research Ethics Committee
PCR: Polymerase chain reaction
PICU: Pediatric intensive care unit
PPROM: Prolonged premature rupture of membrane
UAC: Umbilical artery catheter
UVC: Umbilical vein catheter

## References

[1] Wiens MO, Kumbakumba E, Kissoon N, Ansermino JM, Ndamira A, Larson CP (2012). Pediatric sepsis in the developing world: challenges in defining sepsis and issues in post-discharge mortality. Clin Epidemiol.

[2] Zaidi AKM, Huskins WC, Thaver D, Bhutta ZA, Abbas Z, Goldmann DA (2005). Hospital-acquired neonatal infections in developing countries. Lancet..

[3] United Nations. Interagency Group for Child Mortality Estimation. Levels & Trends in Child Mortality: Report 2010: Estimates Developed by the UN Inter-Agency Group for Child Mortality Estimation [Internet]. UNICEF. New York; 2019 [cited 2020 Jan 3]. https://reliefweb.int/sites/reliefweb.int/files/resources/UN-IGME-Child-Mortality-Report-2019.pdf.

[4] United Nations Children’s Fund. Maternal and Newborn Health Disparities: Nepal [Internet]. UNICEF. New York; 2018 [cited 2020 Jan 6]. https://data.unicef.org/resources/maternal-newborn-health-disparities-country-profiles/Nepal

[5] van der Zwet WC, Kaiser AM, van Elburg RM, Berkhof J, Fetter WPF, Parlevliet GA, Vandenbroucke-Grauls CMJE (2005). Nosocomial infections in a Dutch neonatal intensive care unit: surveillance study with definitions for infection specifically adapted for neonates. J Hosp Infect.

[6] Christina N, Ioanna P, George L, Konstantinos T, Georgios S (2015). Risk factors for nosocomial infections in neonatal intensive care units (NICU). Heal Sci J.

[7] The young infants clinical signs study group (2008). Clinical signs that predict severe illness in children under age 2 months: a multicentre study. Lancet..

[8] Schmutz N, Henry E, Jopling J, Christensen RD (2008). Expected ranges for blood neutrophil concentrations of neonates: the Manroe and Mouzinho charts revisited. J Perinatol.

[9] Bauer A, Kirby W, Sherris J, Turck M (1966). Antibiotic susceptibility testing by a standardized single disk method. Am J Clin Pathol.

[10] CLSI (2017). Performance standards for antimicrobial susceptibility testing.

[11] Thomson KS, Sanders CC (1992). Detection of extended-spectrum β-lactamases in members of the family Enterobacteriaceae: comparison of the double-disk and three-dimensional tests. Antimicrob Agents Chemother.

[12] Woodford N, Fagan EJ, Ellington MJ (2006). Multiplex PCR for rapid detection of genes encoding CTX-M extended-spectrum β-lactamases. J Antimicrob Chemother.

[13] Juan LW, Hying L, Duan GC, Xin ZY, Yin CS, Yang HY (2018). Emergence and mechanism of carbapenem-resistant Escherichia coli in Henan, China, 2014. J Infect Public Health.

[14] Dallenne C, da Costa A, Decré D, Favier C, Arlet G (2010). Development of a set of multiplex PCR assays for the detection of genes encoding important β-lactamases in Enterobacteriaceae. J Antimicrob Chemother.

[15] Poirel L, Walsh TR, Cuvillier V, Nordmann P (2011). Multiplex PCR for detection of acquired carbapenemase genes. Diagn Microbiol Infect Dis.

[16] Nordmann P, Poirel L, Carrër A, Toleman MA, Walsh TR (2011). How to detect NDM-1 producers. J Clin Microbiol.

[17] Woodford N, Ellington MJ, Coelho JM, Turton JF, Ward ME, Brown S (2006). Multiplex PCR for genes encoding prevalent OXA carbapenemases in Acinetobacter spp. Int J Antimicrob Agents.

[18] DeNIS (2016). Characterisation and antimicrobial resistance of sepsis pathogens in neonates born in tertiary care centres in Delhi, India: a cohort study. Lancet Glob Heal.

[19] Ullah O, Khan A, Ambreen, Ahmad I, Akhtar T, Gandapor AJ (2016). Antibiotic sensitivity pattern of bacterial isolates of neonatal septicemia in Peshawar, Pakistan. Arch Iran Med.

[20] Ansari S, Nepal HP, Gautam R, Shrestha S, Neopane P, Chapagain ML (2015). Neonatal septicemia in Nepal: early-onset versus late-onset. Int J Pediatr.

[21] Shah GS, Yadav S, Thapa ASL (2013). Clinical profile and outcome of neonates admitted to neonatal intensive care unit (NICU) at a tertiary Care Centre in Eastern Nepal. J Nepal Paediatr Soc.

[22] Monjur F, Rizwan F, Asaduzzaman M, Nasrin N, Krishna Ghosh N, Sarker Apu A (2010). Antibiotic sensitivity pattern of causative organisms of neonatal septicemia in an urban hospital of Bangladesh. Indian J Med Sci.

[23] Al-Taiar A, Hammoud MS, Cuiqing L, Lee JKF, Lui KM, Nakwan N (2012). Neonatal infections in China, Malaysia, Hong Kong and Thailand. Arch Dis Child Fetal Neonatal Ed.

[24] Tiskumara R, Fakharee SH, Liu CQ, Nuntnarumit P, Lui KM, Hammoud M (2009). Neonatal infections in Asia. Arch Dis Child Fetal Neonatal Ed.

[25] Hafsa A, Fakruddin M, Hakim M, Sharma J (2011). Neonatal bacteremia in a neonatal intensive care unit : analysis of causative organisms and antimicrobial susceptibility. Bangladesh J Med Sci.

[26] Viswanathan R, Singh AK, Mukherjee S, Mukherjee R, Das P, Basu S (2011). Aetiology and antimicrobial resistance of neonatal Sepsis at a tertiary Care Centre in Eastern India: a 3 year study. Indian J Pediatr.

[27] Chaurasia S, Sivanandan S, Agarwal R, Ellis S, Sharland M, Sankar MJ (2019). Neonatal sepsis in South Asia: huge burden and spiralling antimicrobial resistance. BMJ..

[28] Chung The H, Karkey A, Pham Thanh D, Boinett CJ, Cain AK, Ellington M, Baker KS, Dongol S, Thompson C, Harris SR, Jombart T, le Thi Phuong T, Tran Do Hoang N, Ha Thanh T, Shretha S, Joshi S, Basnyat B, Thwaites G, Thomson NR, Rabaa MA, Baker S (2015). A high-resolution genomic analysis of multidrug-resistant hospital outbreaks of Klebsiella pneumoniae. EMBO Mol Med.

[29] Amatya P, Joshi S, Shrestha S (2014). Outbreak of neonatal sepsis outbreak of extended spectrum beta lactamase producing klebsiella species causing neonatal sepsis at Patan hospital in Nepal. J Patan Acad Health Sci.

[30] Ho J, Tambyah PA, Paterson DL (2010). Multiresistant gram-negative infections: a global perspective. Curr Opin Infect Dis.

[31] Russell AB, Sharland M, Heath PT (2012). Improving antibiotic prescribing in neonatal units: time to act. Arch Dis Child - Fetal Neonatal Ed.

[32] Vergnano S (2005). Neonatal sepsis: an international perspective. Arch Dis Child - Fetal Neonatal Ed.

[33] Devi U, Bora R, Das JK, Mahanta J (2018). Extended-spectrum β-lactamase & carbapenemase-producing gram-negative bacilli in neonates from a tertiary care Centre in Dibrugarh, Assam, India. Indian J Med Res.

[34] Breurec S, Bouchiat C, Sire JM, Moquet O, Bercion R, Cisse MF (2016). High third-generation cephalosporin resistant Enterobacteriaceae prevalence rate among neonatal infections in Dakar, Senegal. BMC Infect Dis.

[35] Berezin EN, Solórzano F (2014). Gram-negative infections in pediatric and neonatal intensive care units of Latin America. J Infect Dev Ctries.

[36] Roy S, Gaind R, Chellani H, Mohanty S, Datta S, Singh AK (2013). Neonatal septicaemia caused by diverse clones of Klebsiella pneumoniae & Escherichia coli harbouring blaCTX-M-15. Indian J Med Res.

[37] Datta S, Roy S, Chatterjee S, Saha A, Sen B, Pal T, Som T, Basu S (2014). A five-year experience of Carbapenem resistance in Enterobacteriaceae causing neonatal Septicaemia: predominance of NDM-1. PLoS One.

[38] Yumani DF, van den Dungen FA, van Weissenbruch MM (2013). Incidence and risk factors for catheter-associated bloodstream infections in neonatal intensive care. Acta Paediatr.

[39] Yapicioglu H, Ozcan K, Sertdemir Y, Mutlu B, Satar M, Narli N, Tasova Y (2011). Healthcare-associated infections in a neonatal intensive care unit in Turkey in 2008: incidence and risk factors, a prospective study. J Trop Pediatr.

[40] Dhaneria M, Jain S, Singh P, Mathur A, Lundborg C, Pathak A (2018). Incidence and determinants of health care-associated blood stream infection at a neonatal intensive care unit in Ujjain, India: a prospective cohort study. Diseases.

[41] Ashraf RN, Jalil F, Zaman S, Karlberg J, Khan SR, Lindblad BS (1991). Breast feeding and protection against neonatal sepsis in a high risk population. Arch Dis Child.

[42] Ramasethu J (2017). Prevention and treatment of neonatal nosocomial infections. Matern Heal Neonatol Perinatol.

[43] Kan B, Razzaghian HR, Lavoie PM (2016). An immunological perspective on neonatal Sepsis. Trends Mol Med.

