# Supplementary material for: Risk factors for the development of neonatal sepsis in a neonatal intensive care unit of a tertiary care hospital of Nepal
Source: BMC Infect Dis. 2021 Jun 9;21:546. doi: 10.1186/s12879-021-06261-x (PMC8191200; doi:10.1186/s12879-021-06261-x)
Supplement: Supplementary file 1 — Additional file 1. [file 12879_2021_6261_MOESM1_ESM.docx]

**Risk factors for the development of neonatal sepsis in a neonatal intensive care unit of a tertiary care hospital of Nepal**

Sulochana Manandhar ^1,2^, Puja Amatya ^3^, Imran Ansari ^3^, Niva Joshi ^1^, Nhukesh Maharjan ^1^,

Sabina Dongol ^1^, Buddha Basnyat ^1^, Sameer M. Dixit ^4^, Stephen Baker ^5^ and Abhilasha Karkey ^1*^

^1^ Oxford University Clinical Research Unit, Patan Academy of Health Sciences, Kathmandu, Nepal

^2^ Centre for Tropical Medicine and Global Health, Medical sciences division, Nuffield Department of Medicine, University of Oxford, Linacre College, Oxford, UK

^3^ Department of Pediatrics, Patan Academy of Health Sciences, Patan Hospital, Kathmandu, Nepal

^4^ Center for Molecular Dynamics Nepal, Kathmandu, Nepal

^5^ Cambridge Institute of Therapeutic Immunology & Infectious Disease (CITIID) Department of Medicine, University of Cambridge, Cambridge, UK

***Correspondence**

Dr Abhilasha Karkey

akarkey@oucru.org

**File name: Additional file 1**

File format: .doc

Title of data: Clinical features, risk factors and screening algorithm for sepsis (based on NICU protocol of Patan hospital)

Description of data: Table shows the diagnostic guidelines for neonatal sepsis including clinical features, risk factors and laboratory screening algorithm based on NICU protocol of Patan hospital.

**Additional file 1 Clinical features, risk factors and screening algorithm for sepsis (based on NICU protocol of Patan hospital)**

| Clinical features of sepsis | |
| --- | --- |
| Hypothermia (<35.5˚C) or,  Hyperthermia(>37.7˚C) | Reduced digital capillary refill time |
| Respiratory rate >60 breaths/min | Movement only when stimulated |
| Difficulty in feeding or no feeding | Bulging fontanelle |
| Cyanosis | Mottled skin |
| Grunting | Lethargic |
| Severe chest in-drawing |  |
| Risk Factors for sepsis | |
| Maternal risk factors | **Fetal risk factors** |
| Pre-mature rupture of membrane (>18 hours) | Low birth weight (<2.5 K.G) |
| Fever during late pregnancy | Preterm (<37 weeks of gestation) |
| Urinary tract infection during late pregnancy | Requiring instrumental delivery |
| Sepsis during late pregnancy | Requiring perinatal suctioning |
| Multiple per vaginal examination (>3 times) | Requiring perinatal resuscitation |
| Chorioamnionitis |  |
| Foul smelling vaginal discharge |  |
| Sepsis screen algorithm | |
| Blood | **Abnormal value** |
| Total leukocyte count (TLC) | <7,000 /mm^3^ |
| Absolute neutrophil count (ANC) | Low counts as per Manroe chart |
| Immature/total neutrophil | >0.2 |
| C-reactive protein (CRP) | >6 mg/dl |
| Urine routine microscopy (catheter sample)  *Suggestive of urinary tract infection* | |
| WBC count (in centrifuged sample) | >5 cells/ high-power field |
