# Supplementary material for: Risk factors for the development of neonatal sepsis in a neonatal intensive care unit of a tertiary care hospital of Nepal
Source: BMC Infect Dis. 2021 Jun 9;21:546. doi: 10.1186/s12879-021-06261-x (PMC8191200; doi:10.1186/s12879-021-06261-x)
Supplement: Supplementary file 2 — Additional file 2. [file 12879_2021_6261_MOESM2_ESM.docx]

**Risk factors for the development of neonatal sepsis in a neonatal intensive care unit of a tertiary care hospital of Nepal**

Sulochana Manandhar ^1,2^, Puja Amatya ^3^, Imran Ansari ^3^, Niva Joshi ^1^, Nhukesh Maharjan ^1^,

Sabina Dongol ^1^, Buddha Basnyat ^1^, Sameer M. Dixit ^4^, Stephen Baker ^5^ and Abhilasha Karkey ^1*^

^1^ Oxford University Clinical Research Unit, Patan Academy of Health Sciences, Kathmandu, Nepal

^2^ Centre for Tropical Medicine and Global Health, Medical sciences division, Nuffield Department of Medicine, University of Oxford, Linacre College, Oxford, UK

^3^ Department of Pediatrics, Patan Academy of Health Sciences, Patan Hospital, Kathmandu, Nepal

^4^ Center for Molecular Dynamics Nepal, Kathmandu, Nepal

^5^ Cambridge Institute of Therapeutic Immunology & Infectious Disease (CITIID) Department of Medicine, University of Cambridge, Cambridge, UK

***Correspondence**

Dr Abhilasha Karkey

akarkey@oucru.org

**File name: Additional file 2**

File format: .doc

Title of data: Empirical antimicrobial therapy and duration of antimicrobials used for therapeutic management of neonatal sepsis

Description of data: Table shows the protocol for empirical antimicrobial treatment for neonatal sepsis as practiced in NICU of Patan hospital. The table also shows the data on the use of each listed antimicrobials among the enrolled neonates during this study by number of cases and duration (days) of use.

**Additional file 2 Empirical antimicrobial therapy and duration of antimicrobials used for therapeutic management of neonatal sepsis**

| Antimicrobials | Protocol on empirical antimicrobial therapy | Cases | | Days of use | |
| --- | --- | --- | --- | --- | --- |
|  |  | **Number** | **%** | **Median** | **IQR** |
| Ampicillin | first line | 129 | 90.8 | 6 | 4-8 |
| Amikacin | first line | 128 | 90.2 | 6 | 4-8 |
| Cloxacillin | first line* | 6 | 4.2 | 3.5 | 3-7.8 |
| Cefotaxime | first line** | 80 | 56.3 | 3 | 2-6.3 |
| Chloramphenicol | second line | 57 | 40.1 | 6 | 3-10 |
| Ofloxacin | second line | 60 | 42.3 | 6.5 | 3-10 |
| Meropenem | third line^#^ | 37 | 26.1 | 7 | 7-11 |
| Colistin | third line^#^ | 24 | 16.9 | 10 | 6-12.3 |
| Clindamycin | third line^#^ | 19 | 13.4 | 6 | 3-9.5 |
| Piperacillin-Tazobactam | third line^#^ | 18 | 12.7 | 7 | 4.8-9.8 |
| Fluconazole | third line^#^ | 17 | 11.9 | 7 | 6-12 |
| Vancomycin | third line^#^ | 12 | 8.5 | 7 | 5.3-8.5 |

*added if Gram positive cocci suspected

**added if meningitis suspected

^#^ the third line empirical therapy is constituted of combination of meropenem and ofloxacin; or piperacillin/tazobacam and ofloxacin; or meropenem and vancomycin/linezolid; or cefepime and pipercillin/tazobactam; or meropenem and colistin
