# Supplementary material for: Risk factors for the development of neonatal sepsis in a neonatal intensive care unit of a tertiary care hospital of Nepal
Source: BMC Infect Dis. 2021 Jun 9;21:546. doi: 10.1186/s12879-021-06261-x (PMC8191200; doi:10.1186/s12879-021-06261-x)
Supplement: Supplementary file 3 — Additional file 3. [file 12879_2021_6261_MOESM3_ESM.pdf]

Risk factors for the development of neonatal sepsis in a neonatal intensive care unit of a tertiary care hospital of Nepal

Sulochana Manandhar [1,2], Puja Amatya [3], Imran Ansari [3], Niva Joshi [1], Nhukesh Maharjan [1], Sabina Dongol [1], Buddha Basnyat [1], Sameer M. Dixit [4], Stephen Baker [5] and Abhilasha Karkey [1*]

[1] Oxford University Clinical Research Unit, Patan Academy of Health Sciences, Kathmandu, Nepal
[2] Centre for Tropical Medicine and Global Health, Medical sciences division, Nuffield Department of Medicine, University of Oxford, Linacre College, Oxford, UK
[3] Department of Pediatrics, Patan Academy of Health Sciences, Patan Hospital, Kathmandu, Nepal
[4] Center for Molecular Dynamics Nepal, Kathmandu, Nepal
[5] Cambridge Institute of Therapeutic Immunology & Infectious Disease (CITIID) Department of Medicine, University of Cambridge, Cambridge, UK

***Correspondence**
Dr Abhilasha Karkey
akarkey@oucru.org

File name: Additional file 3

File format: .pdf

Title of data: Overall workflow for sepsis diagnosis

Description of data: The figure depicts details on the workflow used for sepsis diagnosis and the diagnostic outcomes of the neonates that were enrolled in this study

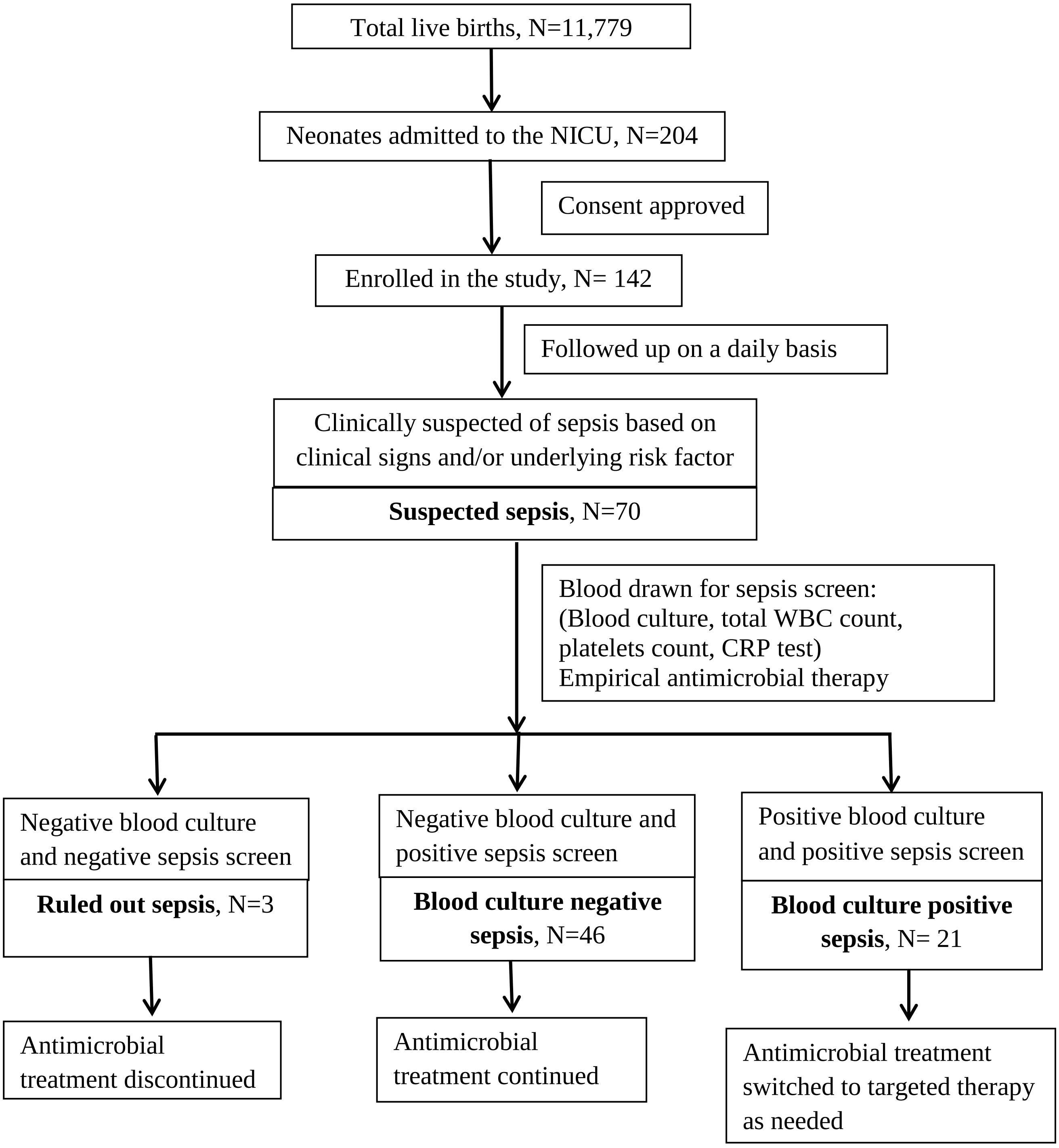
Total live births, N=11,779
Neonates admitted to the NICU, N=204
Consent approved
Enrolled in the study, N= 142
Followed up on a daily basis
Clinically suspected of sepsis based on clinical signs and/or underlying risk factor
Suspected sepsis, N=70
Blood drawn for sepsis screen:
(Blood culture, total WBC count, platelets count, CRP test)
Empirical antimicrobial therapy
Negative blood culture and negative sepsis screen
Ruled out sepsis, N=3
Negative blood culture and positive sepsis screen
Blood culture negative sepsis, N=46
Positive blood culture and positive sepsis screen
Blood culture positive sepsis, N= 21
Antimicrobial treatment discontinued
Antimicrobial treatment continued
Antimicrobial treatment switched to targeted therapy as needed
