# Supplementary material for: Risk factors for the development of neonatal sepsis in a neonatal intensive care unit of a tertiary care hospital of Nepal
Source: BMC Infect Dis. 2021 Jun 9;21:546. doi: 10.1186/s12879-021-06261-x (PMC8191200; doi:10.1186/s12879-021-06261-x)
Supplement: Supplementary file 4 — Additional file 4. [file 12879_2021_6261_MOESM4_ESM.docx]

**Risk factors for the development of neonatal sepsis in a neonatal intensive care unit of a tertiary care hospital of Nepal**

Sulochana Manandhar ^1,2^, Puja Amatya ^3^, Imran Ansari ^3^, Niva Joshi ^1^, Nhukesh Maharjan ^1^,

Sabina Dongol ^1^, Buddha Basnyat ^1^, Sameer M. Dixit ^4^, Stephen Baker ^5^ and Abhilasha Karkey ^1*^

^1^ Oxford University Clinical Research Unit, Patan Academy of Health Sciences, Kathmandu, Nepal

^2^ Centre for Tropical Medicine and Global Health, Medical sciences division, Nuffield Department of Medicine, University of Oxford, Linacre College, Oxford, UK

^3^ Department of Pediatrics, Patan Academy of Health Sciences, Patan Hospital, Kathmandu, Nepal

^4^ Center for Molecular Dynamics Nepal, Kathmandu, Nepal

^5^ Cambridge Institute of Therapeutic Immunology & Infectious Disease (CITIID) Department of Medicine, University of Cambridge, Cambridge, UK

***Correspondence**

Dr Abhilasha Karkey

akarkey@oucru.org

**File name: Additional file 4**

File format: .doc

Title of data: Details of multiplex PCR for detection of resistance gene markers of ESBL and carbapenemase production

Description of data: The table lists the name and nucleotide sequence of the PCR primers along with specific thermocycler conditions (final primer concentration, annealing temperature and PCR product size) for each PCR targets on genes conferring antimicrobial resistance to beta-lactam and carbapenem antimicrobials.

**Additional file 4 Details of multiplex PCR for detection of resistance gene markers of ESBL and carbapenemase production**

| Genes | Primers | Primer sequence (5' - 3') | Primer final conc. | Anneal °C/sec | Product size (bp) | |
| --- | --- | --- | --- | --- | --- | --- |
| bla_CTX-M1_ | CTX-M1F | AAAAATCACTGCGCCAGTTC | 0.1uM | 60°C/  40 sec | 415 |  |
|  | CTX-M1R | AGCTTATTCATCGCCACGTT | 0.1uM |  |  |  |
| bla_CTX-M2_ | CTX-M2F | CGACGCTACCCCTGCTATT | 0.1uM |  | 552 |  |
|  | CTX-M2R | CCAGCGTCAGATTTTTCAGG | 0.1uM |  |  |  |
| bla_CTX-M9_ | CTX-M9F | CAAAGAGAGTGCAACGGATG | 0.1uM |  | 205 |  |
|  | CTX-M9R | ATTGGAAAGCGTTCATCACC | 0.1uM |  |  |  |
| bla_CTX-M8/25_ | CTX-M8F | TCGCGTTAAGCGGATGATGC | 0.1uM |  | 666/  327 |  |
|  | CTX-M25F | GCACGATGACATTCGGG | 0.1uM |  |  |  |
|  | CTX-M8/25R | AACCCACGATGTGGGTAGC | 0.2uM |  |  |  |
| bla_TEM_ | TEM F | TGCGGTATTATCCCGTGTTG | 0.1uM | 57°C/  40 sec | 300 |  |
|  | TEM R | TCGTCGTTTGGTATGGCTTC | 0.1uM |  |  |  |
| bla_SHV_ | MultiTSO-S_F | AGCCGCTTGAGCAAATTAAAC | 0.2uM |  | 713 |  |
|  | MultiTSO-S_R | ATCCCGCAGATAAATCACCAC | 0.2uM |  |  |  |
| bla_OXA_ | MultiTSO-O_F | GGCACCAGATTCAACTTTCAAG | 0.2uM |  | 564 |  |
|  | MultiTSO-O_R | GACCCCAAGTTTCCTGTAAGTG | 0.2uM |  |  |  |
| bla_KPC_ | KPC-F | CGTCTAGTTCTGCTGTCTTG | 0.2uM | 52°C/  40 sec | 798 |  |
|  | KPC-R | CTTGTCATCCTTGTTAGGCG | 0.2uM |  |  |  |
| bla_NDM1_ | NDM-1F | GGTTTGGCGATCTGGTTTTC | 0.2uM |  | 621 |  |
|  | NDM-1R | CGGAATGGCTCATCACGATC | 0.2uM |  |  |  |
| bla_OXA48_ | OXA-48-F | GCGTGGTTAAGGATGAACAC | 0.2uM |  | 438 |  |
|  | OXA-48-R | CATCAAGTTCAACCCAACCG | 0.2uM |  |  |  |
| bla_OXA23_ | OXA-23-likeF | GATCGGATTGGAGAACCAGA | 0.2uM | 52°C/  40 sec | 501 |  |
|  | OXA-23-likeR | ATTTCTGACCGCATTTCCAT | 0.2uM |  |  |  |
| bla_OXA24_ | OXA-24-likeF | GGTTAGTTGGCCCCCTTAAA | 0.2uM |  | 246 |  |
|  | OXA-24-likeR | AGTTGAGCGAAAAGGGGATT | 0.2uM |  |  |  |
| bla_OXA51_ | OXA-51-likeF | TAATGCTTTGATCGGCCTTG | 0.2uM |  | 353 |  |
|  | OXA-51-likeR | TGGATTGCACTT CAT CTT GG | 0.2uM |  |  |  |
| bla_OXA58_ | OXA-58-likeF | AAGTATTGGGGCTTGTGCTG | 0.2uM |  | 599 |  |
|  | OXA-58-likeR | CCCCTCTGCGCTCTACATAC | 0.2uM |  |  |  |
| bla_NDM1_ | NDM-1F | GGTTTGGCGATCTGGTTTTC | 0.2uM | 61°C/  40 sec | 621 |  |
|  | NDM-1R | CGGAATGGCTCATCACGATC | 0.2uM |  |  |  |
