# Supplementary material for: Risk factors for the development of neonatal sepsis in a neonatal intensive care unit of a tertiary care hospital of Nepal
Source: BMC Infect Dis. 2021 Jun 9;21:546. doi: 10.1186/s12879-021-06261-x (PMC8191200; doi:10.1186/s12879-021-06261-x)
Supplement: Supplementary file 5 — Additional file 5. [file 12879_2021_6261_MOESM5_ESM.docx]

**Risk factors for the development of neonatal sepsis in a neonatal intensive care unit of a tertiary care hospital of Nepal**

Sulochana Manandhar ^1,2^, Puja Amatya ^3^, Imran Ansari ^3^, Niva Joshi ^1^, Nhukesh Maharjan ^1^,

Sabina Dongol ^1^, Buddha Basnyat ^1^, Sameer M. Dixit ^4^, Stephen Baker ^5^ and Abhilasha Karkey ^1*^

^1^ Oxford University Clinical Research Unit, Patan Academy of Health Sciences, Kathmandu, Nepal

^2^ Centre for Tropical Medicine and Global Health, Medical sciences division, Nuffield Department of Medicine, University of Oxford, Linacre College, Oxford, UK

^3^ Department of Pediatrics, Patan Academy of Health Sciences, Patan Hospital, Kathmandu, Nepal

^4^ Center for Molecular Dynamics Nepal, Kathmandu, Nepal

^5^ Cambridge Institute of Therapeutic Immunology & Infectious Disease (CITIID) Department of Medicine, University of Cambridge, Cambridge, UK

***Correspondence**

Dr Abhilasha Karkey

akarkey@oucru.org

**File name: Additional file 5**

File format: .doc

Title of data: The demographic summary of neonatal, maternal and environmental features for 142 enrolled neonates

Description of data: The table shows several characteristics pertaining to neonatal sepsis including maternal and neonatal demographic features, neonatal laboratory parameters, therapeutic/supportive clinical care, features on neonatal feeding and the final outcome of the neonates. These characteristics were also used in statistical analyses for investigating the risk factors for neonatal sepsis among enrolled neonates.

**Additional file 5 The demographic summary of neonatal, maternal and environmental features for 142 enrolled neonates.**

| Features | n or median | % or IQR |
| --- | --- | --- |
| Neonatal features |  |  |
| Birth weight (grams) | 2250 | 2300-2700 |
| Low birth weight (1500-2500 grams) | 62 | 43.7 |
| Very birth weight (<1500 grams) | 22 | 15.5 |
| Gestational age (weeks) | 36 | 34.7-35 |
| Preterm (<37 weeks of gestation) | 83 | 58.5 |
| Male | 93 | 65.5 |
| Obstetric and peri-partum features |  |  |
| Premature rupture of membrane for > 18 hours (PROM) | 34 | 23.9 |
| Number of vaginal examination, 3 or more times | 10 | 7.0 |
| Culture positive urinary tract infection in late pregnancy | 6 | 4.2 |
| Culture positive high vaginal infection in late pregnancy | 4 | 2.8 |
| Mode of delivery |  |  |
| Assisted or instrumental delivery | 14 | 9.9 |
| Vaginal delivery | 31 | 21.8 |
| C-section delivery | 96 | 67.6 |
| Post-partum fever | 15 | 10.6 |
| Characteristics of amniotic fluid |  |  |
| Meconium stained | 21 | 14.8 |
| Clear | 116 | 81.7 |
| Foul smelling | 0 | 0.0 |
| Fetal distress | 20 | 14 |
| Suctioning at birth | 60 | 42.2 |
| Resuscitation at birth | 26 | 18.3 |
| Intubation at birth | 22 | 15.5 |
| Laboratory features of blood |  |  |
| Total WBC count in blood (X 10^3^/µl) | 10.9 | 7 - 15.9 |
| Leukopenia (<7,000 WBC/µl) | 92 | 24.3 |
| Leukocytosis (>30,000 WBC /µl) | 19 | 5.0 |
| Total platelets count (X 10^3^/µl) | 191 | 124.5 - 265 |
| Thrombocytopenia (<150,000 platelets/µl) | 125 | 33.7 |
| C-reactive protein (CRP) level (mg/dl) | 9 | 2.5 - 39 |
| Elevated CRP (>6 mg/dl) | 99 | 58.6 |
| Modalities of therapeutic /supportive care | | |
| Use of ionotropes | 67 | 47.2 |
| Blood transfusion | 52 | 36.6 |
| Insertion of invasive devices | | |
| Central line types: |  |  |
| Umbilical artery catheter (UAC) | 50 | 35.2 |
| Umbilical vein catheter (UVC) | 59 | 41.5 |
| Intravenous cannula | 124 | 87.3 |
| Respiratory support types: |  |  |
| Mechanical ventilation | 105 | 73.9 |
| CPAP | 113 | 79.6 |
| Nasal prong | 50 | 35.2 |
| Spontaneous | 27 | 19.0 |
| Foley's catheter | 61 | 42.9 |
| Dwelling time of invasive devices (days) | | |
| *Central line types:* |  |  |
| Umbilical artery catheter (UAC) | 6 | 5-8 |
| Umbilical vein catheter (UVC) | 6 | 4-8 |
| Intravenous cannula | 11 | 7-16 |
| *Respiratory support types:* |  |  |
| Mechanical ventilation | 5 | 3-10 |
| CPAP | 3 | 2-4 |
| Nasal prong | 1 | 1-2 |
| Spontaneous | 2 | 1-3 |
| Foley's catheter | 6 | 5-10 |
| Neonatal feeding features: | | |
| Never breast fed * | 120 | 85 |
| Ever breast fed * | 20 | 14.2 |
| Never spoon fed * | 116 | 81.7 |
| Ever spoon fed * | 24 | 16.9 |
| Never fed enterally* | 23 | 16.2 |
| Ever fed enterally* | 117 | 82.4 |
| Days not breastfed | 7.5 | 4-12 |
| Days not spoon-fed | 8 | 4-12.2 |
| Days enterally-fed | 4 | 2-7 |
| Outcome |  |  |
| NICU stay >7 days | 65 | 45.8 |
| NICU stay >14 days | 22 | 15.5 |
| Hospital stay >14 days | 53 | 37.3 |
| Days of NICU stay | 7 | 4-12 |
| Days of hospital stay | 16 | 10-26 |
| Final outcome |  |  |
| Survived | 101 | 71.1 |
| Dead | 20 | 14.1 |
| Left against medical advice | 4 | 2.8 |

CPAP, Continuous Positive Airway Pressure

* The cases were mutually exclusive
